# Comparative Study on Kinetics of Ethylene and Propylene Polymerizations with Supported Ziegler–Natta Catalyst: Catalyst Fragmentation Promoted by Polymer Crystalline Lamellae

**DOI:** 10.3390/polym11020358

**Published:** 2019-02-19

**Authors:** Zhen Zhang, Baiyu Jiang, Feng He, Zhisheng Fu, Junting Xu, Zhiqiang Fan

**Affiliations:** MOE Key Laboratory of Macromolecular Synthesis and Functionalization, Department of Polymer Science and Engineering, Zhejiang University, Hangzhou 310027, China; 11329008@zju.edu.cn (Z.Z.); jiangbaiyu827@yeah.net (B.J.); 11329007@zju.edu.cn (F.H.); fuzs@zju.edu.cn (Z.F.); xujt@zju.edu.cn (J.X.)

**Keywords:** ethylene, propylene, Ziegler–Natta catalyst, kinetics, morphology, catalyst fragmentation

## Abstract

The kinetic behaviors of ethylene and propylene polymerizations with the same MgCl_2_-supported Ziegler–Natta (Z–N) catalyst containing an internal electron donor were compared. Changes of polymerization activity and active center concentration ([C*]) with time in the first 10 min were determined. Activity of ethylene polymerization was only 25% of that of propylene, and the polymerization rate (*R*_p_) quickly decayed with time (*t*_p_) in the former system, in contrast to stable *R*_p_ in the latter. The ethylene system showed a very low [C*]/[Ti] ratio (<0.6%), in contrast to a much higher [C*]/[Ti] ratio (1.5%–4.9%) in propylene polymerization. The two systems showed noticeably different morphologies of the nascent polymer/catalyst particles, with the PP/catalyst particles being more compact and homogeneous than the PE/catalyst particles. The different kinetic behaviors of the two systems were explained by faster and more sufficient catalyst fragmentation in propylene polymerization than the ethylene system. The smaller lamellar thickness (<20 nm) in nascent polypropylene compared with the size of nanopores (15–25 nm) in the catalyst was considered the key factor for efficient catalyst fragmentation in propylene polymerization, as the PP lamellae may grow inside the nanopores and break up the catalyst particles.

## 1. Introduction

After rapid and continuous growth in the last sixty years, the industrial production of polyolefins (polyethylene, polypropylene, and olefin copolymers) has become one of the most important branches of modern chemical industry, and MgCl_2_-supported Ziegler–Natta (Z–N) catalysts are playing dominant roles in polyolefin production. Despite extensive fundamental studies in this field over the past decades, there are still many unsolved problems concerning the polymerization mechanism and relationships between catalyst structure and polymerization behaviors. The kinetics and mechanism of ethylene and propylene polymerizations with Z–N catalysts have been studied in a broad span of reaction durations ranging from less than 1 s to more than one hour [1,2,3,4,5,6,7,8,9,10,11,12,13,14,15,16,17,18,19,20,21,22,23]. When ethylene and propylene polymerizations with the same catalyst are compared, several peculiarities in the reaction kinetics have been reported. Y.V. Kissin reported that ethylene polymerization with a TiCl_3_-based classical Z–N catalyst and MgCl_2_-supported Z–N catalyst both presented build-up type rate curves with a rather long induction period (10–30 min), in which the reaction rate gradually grew to the stationary level. However, propylene polymerization with the same catalyst presented decay type rate curves, in which the reaction rate quickly rose to the maximum and then began descending over a long period [19,20]. Considering the similar chain initiation and propagation mechanisms of the two monomers and the very fast formation process of the polymerization active centers, it is hard to explain such different kinetic behaviors by a unified mechanistic model [16,24]. It is even more puzzling that ethylene polymerization activity was much lower than that of propylene polymerization when some supported Z–N catalysts containing an internal electron donor were used [25,26]. The activity of ethylene polymerization was found to be markedly enhanced by introducing a small amount of propylene before ethylene (so-called prepolymerization) [25,27,28]. Because the intrinsic reactivity of ethylene polymerization is evidently higher than that of propylene on the same catalyst [22,25], it is hard to attribute such a phenomenon to certain kinds of chemical activation effect.

V.A. Zakharov et al. studied ethylene and propylene polymerizations with a Ti-based supported Z–N catalyst containing a dibutylphthalate internal donor [25]. They found that the fraction of active centers ([C*]/[Ti]; here, C* denotes active center) was larger in ethylene polymerization than in propylene polymerization, and chain propagation rate constants (*k*_p_) were also larger in the former. The ethylene polymerization activity was markedly enhanced by using a similar catalyst with higher porosity; meanwhile, the activity of the propylene polymerization was not very sensitive to catalyst porosity. The occurrence of diffusion limitation in ethylene polymerization was proposed as the reason for its sensitivity to catalyst porosity. In our previous works, significant enhancement of [C*]/[Ti] with reaction time in the initial stage of ethylene (co)polymerization was observed, and fragmentation of the catalyst particles by hydraulic force of the growing polymer chains was considered the reason for the enhancement of active center numbers [21,22]. According to these results, fragmentation of the catalyst particles significantly influences the [C*]/[Ti] fraction in the initial stage and its changes with time, and thus determines the polymerization kinetics. To explicitly disclose origins of the different reaction kinetics of ethylene and propylene polymerizations, it is thus necessary to directly compare the [C*]/[Ti] versus time profiles of the two systems.

In this work, the microkinetics of ethylene and propylene polymerizations with a supported Z–N catalyst containing a phthalate type internal donor was systematically studied, including tracing the changes of [C*]/[Ti] in the polymerization processes and characterizing the structure of nascent polymer/catalyst particles formed in different reaction periods. The method of counting active centers by quench-labeling the propagation chains with 2-thiophenecarbonyl chloride (TPCC) was used. This method was developed in our laboratory and applied to various olefin polymerization systems catalyzed by both heterogeneous and homogeneous catalysts [21,22,23,29,30,31,32,33,34]. The collected results provide new evidence that clearly shows the crucial importance of catalyst particle fragmentation in determining the polymerization kinetics and catalyst efficiency. The knowledge produced through the research can promote an in-depth understanding of olefin polymerization kinetics and mechanism, and guide further optimization of the polymerization process, as well as catalyst development.

## 2. Materials and Methods

### 2.1. Reagents

A commercial supported Z–N catalyst TiCl_4_/Di/MgCl_2_ (Ti content = 2.7 wt%, Di = dibutylphthalate, provided by SINOPEC Group, Nanjing, China) was used for the polymerization. Triethylaluminum (TEA) was purchased from Albemarle (Charlotte, NC, USA) and used as 2M solution in *n*-heptane. 2-thiophenecarbonyl chloride (TPCC, >98%), purchased from Alfa Aesar Co. (Shanghai, China), was distilled and diluted to 2M solution in *n*-heptane before use. *n*-Heptane was dried over 4 A molecular sieves under dry nitrogen and refluxed over Na before use. Ethylene and propylene (polymerization grade, supplied by Minxing Gas Co., Hangzhou, China) were purified by molecular sieves and manganese-based deoxygen agent in a gas purification system made by Dalian Samat Chemicals Co., Ltd. (Dalian, China). All other chemicals were obtained commercially and used without further purification unless otherwise stated.

### 2.2. Polymerization and Quenching Reaction

All operations were carried out under a dry nitrogen atmosphere using standard Schlenk line or glove box techniques. Polymerization runs were conducted in 250 mL glass reactor equipped with a magnetic stirrer and gas inlet, which was immersed in thermostat bath of 40 °C. After evacuating the reactor and refilling it with monomer gas (ethylene or propylene) three times, the planned amount of *n*-heptane was added to the reactor under the monomer atmosphere. Then, the TEA solution (Al/Ti = 40) was added, and the calculated amount of catalyst was flushed to the reactor by *n*-heptane to launch the polymerization. Ethylene or propylene flow of 1 atm pressure was continuously supplied to the reactor for the stipulated polymerization time (*t*_p_). In the experiments, for the kinetic study, TPCC (TPCC/Al = 2.5) was quickly injected into the reactor after the designed *t*_p_ in order to quench the polymerization. After 5 min of quenching reaction, acidified ethanol was added to decompose the catalyst and quencher, and the produced polymer was precipitated with an excess of ethanol. The collected polymer samples were further purified according to the procedures mentioned in our previous work to remove all sulfur-containing impurities [29,30]. In the experiments, for studying morphology (SEM observation) and pore size distribution (Brunauer–Emmett–Teller (BET) analysis) of the polymer/catalyst particles, when the calculated *t*_p_ was reached, the reactor was quickly immersed in liquid nitrogen to stop the reaction, and then CO_2_ was bubbled to the reactor to convert TEA into unreactive chemicals, so as to preserve the morphology of nascent catalyst/polymer particles. The particles were vacuum dried at room temperature after removing the solvent, and then stored under nitrogen for SEM and BET analysis.

### 2.3. Characterization

The sulfur content of the quenched polymer was measured with a YHTS-2000 fluorescence UV sulfur analyzer (Jiangyan Yinhe Instrument Co., Jiangyan, China, detection limit = 0.05 ppm). The polymer sample for the analysis was solid powder (2–4 mg, weighed to ± 0.01 mg), and the average value of three parallel measurements was recorded for each sample.

Molecular weight and molecular weight distribution of the polymer samples were measured by gel permeation chromatography (GPC) in a PL 220 GPC instrument (Polymer Laboratories, Shropshire, UK) with three PL mixed B columns (500 ~ 10^7^) at 150 °C in 1,2,4-trichlorobenzene. Universal calibration against polystyrene standards was adopted.

Differential scanning calorimetry (DSC) analysis of the polymers was conducted using a TA Q200 DSC instrument under N_2_ atmosphere. Then, 2–3 mg of each sample was sealed in an aluminum sample pan, and melting endotherm in the first heating scan was recorded at a heating rate of 10 °C/min by scanning from 40 to 180 °C.

Scanning electron microscope observations of the PE and PP particles were made with a Hitachi-4800 SEM. Micrographs was taken at 3 kV acceleration voltage. Before SEM observations, all the sample surfaces were vacuum sputtered with a thin layer of gold.

Nitrogen adsorption–desorption isotherms and pore-size distributions of the polymer particles were measured using an AUTOSORB-1-C instrument (Quantachrome, Boynton Beach, FL, USA) at 77 K. Prior to the experiments, the samples were degassed in vacuum at 90 °C for 24 h. Their specific surface areas were determined on the basis of the BET (Brunauer–Emmett–Teller) adsorption model. The total pore volumes and average pore sizes were also calculated. The pore size distributions were statistically obtained by using a Quantachrome software following BJH theory according to the desorption isotherms.

## 3. Results and Discussion

### 3.1. Polymerization Kinetics

**Figure 1:**
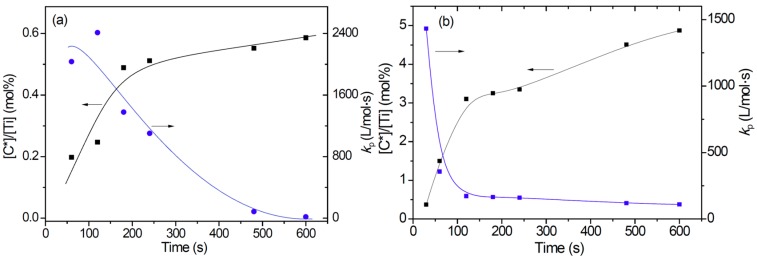
(**a**) Influence of polymerization time on the fraction of active centers and apparent propagation rate constant of ethylene polymerization; (**b**) influence of polymerization time on the fraction of active centers and apparent propagation rate constant of propylene polymerization.

A series of ethylene polymerization, as well as propylene polymerization, was conducted under the same conditions for different durations, and each polymerization run was quenched by adding TPCC when the planned reaction time was reached. The reaction products were analyzed for sulfur content, and the fraction of active centers ([C*]/[Ti]) of each polymer sample was determined based on its sulfur content. The polymerization rate *R*_p_ was determined from differentiation of the curve of polymer yield versus time. According to the rate equation *R*_p_ = *k*_p_[C*][M], which has been well established for most catalyzed olefin polymerizations, the apparent chain propagation rate constant *k*_p_ was calculated using the *R*_p_ and [C*]/[Ti] data, as well as equilibrium monomer concentration in the reaction system. GPC analysis on the polymer samples was also carried out to determine their molecular weight distribution and average molecular weight. The results are shown in Table 1 and Figure 1, respectively.

It is seen that the activity of ethylene polymerization was noticeably lower than that of propylene polymerization in the 10 min reaction period. The former system experienced a moderate rise of reaction rate in the first 3 min, but then the rate quickly decreased and fell to nearly zero activity after 10 min. In contrast, the propylene system showed a much higher reaction rate at the very beginning and maintained it in the following 10 min. As shown in Figure 1, the lower activity of ethylene polymerization in the first 4 min can be mainly attributed to its lower fraction of active centers compared with that of propylene polymerization. In the later stage (*t*_p_ = 6–10 min), the *k*_p_ value of ethylene polymerization decreased to very low level, resulting in even lower polymerization rate.

**Figure 2:**
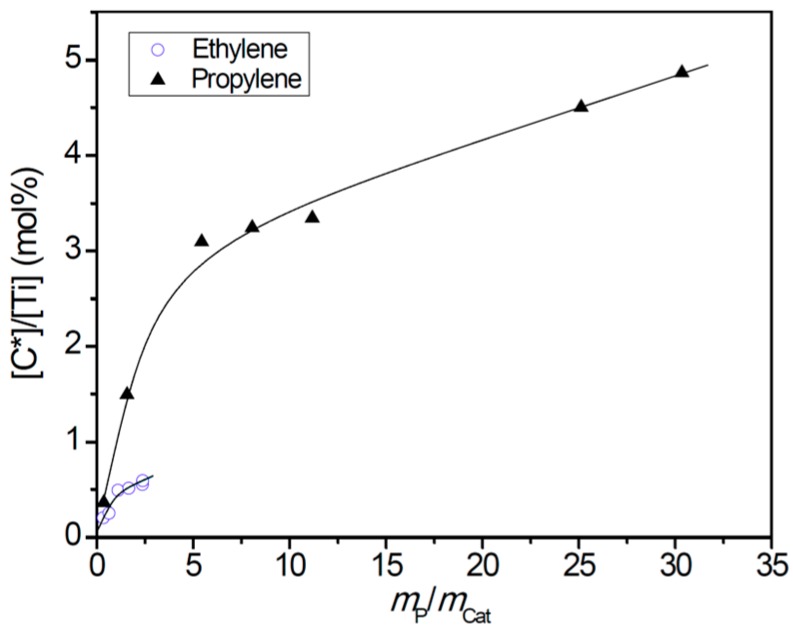
Changes of the fraction of active centers with polymer/catalyst mass ratio in ethylene and propylene polymerizations.

In our previous study on ethylene polymerization kinetics with an industrial supported Z–N catalyst designed for polyethylene production, similar growth of [C*]/[Ti] with polymerization time was observed, and the growth of [C*]/[Ti] was correlated with gradual disintegration of the catalyst particles in the reaction process [21,22,33]. Hydraulic forces of the growing polymer phase were considered as the dominant factor in the particle fragmentation. By comparing the changes of [C*]/[Ti] with mass ratio of polymer to catalyst (*m*_P_/*m*_Cat_) in the two polymerization systems (see Figure 2), it is seen that both systems showed a two-stage increase of [C*]/[Ti] with *m*_P_/*m*_Cat_, where the first stage showed rapid growth of [C*]/[Ti], which slowed down but continued in the second stage. The main difference between the two systems was the much higher efficiency of particle fragmentation by polymer in the propylene system than that of the ethylene system. This means that the same amount of polymer in the ethylene system caused particle fragmentation to a much lesser extent than the propylene polymerization.

The rapid decrease of *k*_p_ value in the polymerization processes can be explained by the increase of diffusion barrier with the increase of *m*_P_/*m*_Cat_ ratio, because the polymer layer surrounding the catalyst fragments where the active centers are anchored can retard mass transfer inside the polymer/catalyst particles, and reduce local monomer concentration in the particles [35,36,37,38]. For this reason, it can be assumed that the *k*_p_ value is reversely proportional to the *m*_P_/*m*_Cat_ ratio. In fact, such correlation does exist when the *m*_P_/*m*_Cat_ ratio increased from 0 to about 2 (see Table 1 and Figure S1 of Supplementary Materials). In propylene polymerization, the *k*_p_ value gradually leveled off after about 2 min of polymerization, but in ethylene polymerization, the *k*_p_ value decayed continuously until reaching zero.

The weight average molecular weight of polyethylene evidently increased with reaction time, which can be explained by the decay of a part of active centers producing low molecular weight polymer [19]. However, the molecular weight of polypropylene was kept nearly constant during the reaction period.

### 3.2. Morphology of the Polymer/Catalyst Particles

**Figure 3:**
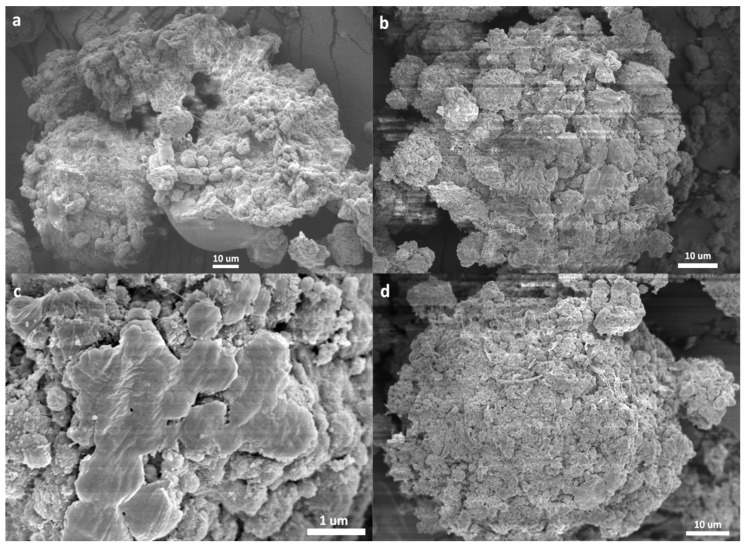

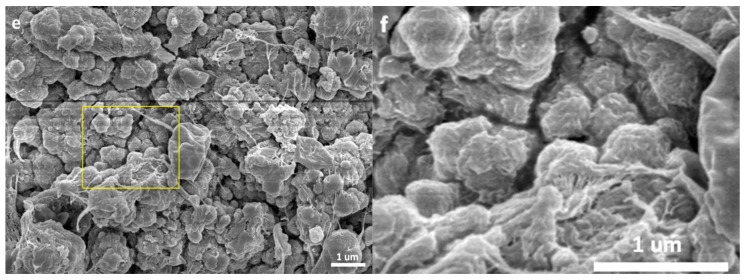
SEM pictures of PE/catalyst particles formed at different polymerization times: (**a**) 60 s; (**b**,**c**) 120 s; (**d**–**f**) 180 s (samples E2, E3, and E4 in Table 1. The enlarged picture of the marked area in (**e**) is shown in (**f**)).

**Figure 4:**
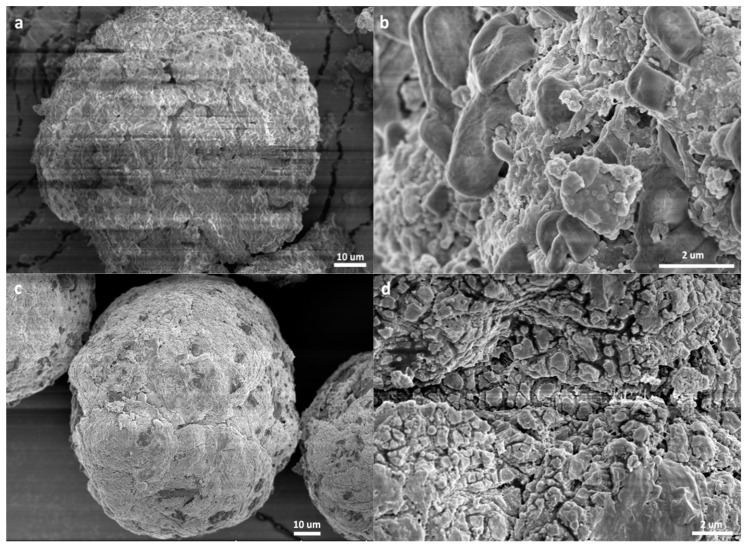

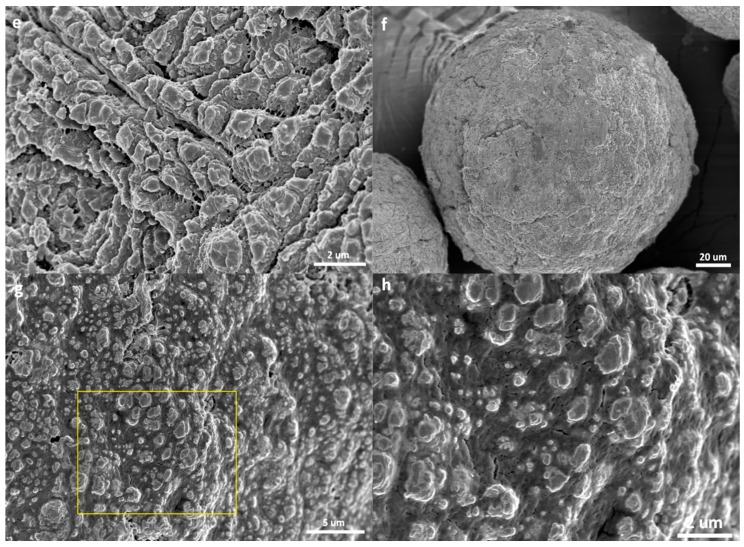
SEM pictures of PP/catalyst particles formed at different polymerization times: (**a**,**b**) 30 s; (**c**,**d**) 60 s; (**e**) 120 s; (**f**–**h**) 180 s (samples P1, P2, P3, and P4 in Table 1).

The remarkably different kinetic behaviors of ethylene and propylene polymerizations prompted us to search for more evidence that can disclose the mechanism behind the phenomena. As discussed above, the changes of [C*]/[Ti] and *k*_p_ in the polymerization process are all closely related with the polymer/catalyst particles, because these are the actual places in which the reactions take place. Research into the morphology of nascent polymer/catalyst particles may provide important information on the polymerization process. Several experimental methods have been reported in such studies. Di Martino et al. developed a quenched-flow method, in which olefin polymerization of very short duration is “quenched” with a poison without destroying the polymer/catalyst particles [39]. T. Taniike et al. used a similar method to fix the nascent morphology of the particles and trace their development in the polymerization process [40]. In this work, a method similar to that adopted by M. Terano et al. [41] was used to collect nascent polymer/catalyst particles and condition them for SEM observation. Typical SEM pictures of PE/catalyst and PP/catalyst particles collected at different polymerization times are shown in Figure 3 and Figure 4, respectively. More SEM pictures of the polymer/catalyst particles are shown in Figures S3 and S4 of Supplementary Materials. SEM pictures of the original catalyst particles are shown in Figure S2 of Supplementary Materials.

As shown in Figure S2, the catalyst particles have a regular spherical shape with a diameter ranging from 10 to 50 µm. The enlarged picture of the internal part of a particle showed that the whole particle was composed of sub-particles of 150–500 nm in size, which are clearly divided by tiny cracks between them. Totally speaking, the catalyst particle has a rather compact solid structure, besides the presence of broad cracks (width > 0.1 µm) on the outer surface and the internal part. 

When ethylene polymerization was conducted for short time, the nascent PE/catalyst particles presented a rather loose and porous morphology, and a part of spherical particles was seriously broken into irregular fragments. When the polymerization was extended to 3 min, the roughly spherical particles can be found to be composed of irregular small particles of about 0.3–1.5 µm. By comparing with the sub-particles in the original catalyst, and considering the rather low PE/catalyst mass ratio (1.12 at *t*_p_ = 180 s), it is likely that these small particles are composites of PE that cover the sub-particles, where the PE chains were formed by active centers of the sub-particles. Platelet structures in the small particles (see Figure 3f) can be attributed to PE crystalline lamellae.

The morphology of PP/catalyst particles (Figure 4) was noticeably different from that of PE/catalyst particles. As propylene polymerization proceeded from 0 to 3 min, the spherical shape of the catalyst particles was well preserved. Similar phenomena have been well reported in the past [42,43,44,45,46]. After only 30 s of polymerization, the particle’s outer surface looked like a mixture of tiny irregular polymer particles and large (1 to 2 µm) grains with smooth surfaces. The latter could be the solid phase of the catalyst, or an inorganic component covered by a thin polymer layer. As the polymerization continued to 60–120 s, most of these micrometer-sized hard particles disappeared, meaning that they were disintegrated by the growing PP chains. Though there are domains with sizes of 0.5 to 2 µm on the outer surface, they were intimately interconnected with each other. As the polymerization proceeded for 180 s, the outer surface became smoother. If the micrometer-sized plateaus on the particle’s outer surface seen at *t*_p_ = 180 s were originated from the sub-particles of the catalyst granule, merging of their boundaries means that a large proportion of the catalyst’s sub-particles underwent severe disintegration in the later stage of polymerization. By comparing Figure 3d–f and Figure 4f–h, it is clear that the PE/catalyst particles are loose aggregates of sub-particles of about 0.3–1.5 µm, while the sub-particles in PP/catalyst particles are intimately merged with each other.

**Figure 5:**
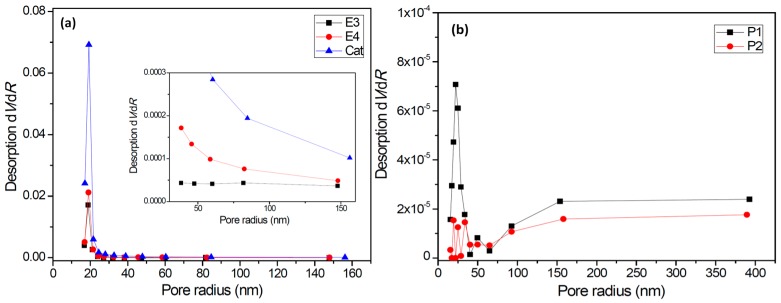
Pore size distributions of the catalyst and the polymer/catalyst particles. (**a**) Polyethylene/catalyst and catalyst and (**b**) polypropylene/catalyst.

Nitrogen adsorption–desorption isotherms of the catalyst and typical polymer/catalyst nascent particles (E3, E4, P1, and P2) were measured to determine their pore size distribution curves (see Figure 5 and Figure S5 of Supplementary Materials). As seen in Figure 5a, the catalyst particles presented a strong peak at about 20 nm in the pore size distribution. Because the sub-particles (150–500 nm) in the catalyst are much larger than 20 nm, it is likely that these tiny pores are mainly located inside the sub-particles. In the PE/catalyst particles, the number of pores around 20 nm was noticeably reduced to 1/3–1/4 of those in the catalyst, but the number of larger pores (>50 nm) was reduced further. This is especially evident in sample E3, which has a PE/catalyst mass ratio of only 0.645. A possible explanation is that most large pores with size >50 nm were filled up by the PE chains, but only a relatively smaller proportion of 20 nm pores were filled up after ethylene polymerization.

In sharp contrast with the PE/catalyst particles, in the PP/catalyst particles, the volume of the 20 nm pores was reduced to only about 1/1000 of that in the catalyst particles when *m*_P_/*m*_Cat_ was only 0.36 (sample P1), and it was further reduced to nearly zero when *m*_P_/*m*_Cat_ increased to 1.56 (sample P2). The volume of larger pores in the PP/catalyst particles was also noticeably smaller than that in the PE/catalyst particles and the original catalyst. As shown in Table 2, the specific surface area and total pore volume decreased in the following order: catalyst particle > PE/catalyst particle >> PP/catalyst particle; meanwhile, the average pore size presented a reversed order. The PP/catalyst particles are far less porous than the PE/catalyst particles. This could be correlated to the higher [C*]/[Ti] ratio reached in propylene polymerization than in ethylene polymerization. The greater extent of catalyst fragmentation in the former system could have caused the disappearance of most of the 20 nm pores, and released more active sites on the exposed surfaces. PP chains produced by these active sites can than cover the exposed surfaces, forming rather compact polymer/catalyst particles.

### 3.3. Polymer Aggregation State in Nascent Polymer Particle

**Figure 6:**
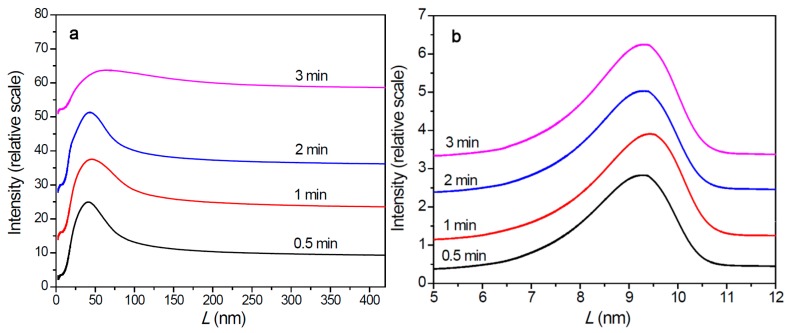
Lamellar thickness distribution of nascent polymer collected at different polymerization time. (**a**) Polyethylene and (**b**) polypropylene.

The aggregation state of the just-formed polymer phase was analyzed by recording the first DSC heating scan on nascent polymer particles that had not been heated to melting before. As shown in Table 3 and Figure S6 of the Supplementary Materials, PE in the nascent polymer particles had a rather high melting temperature and crystallinity, while the melting temperature of the nascent PP samples was not much different from that of PP crystallized from melt. The melting temperature of the PE sample was noticeably higher than that of the same sample crystallized from melt (see Table S1 of the Supplementary Materials). Lamellar thickness distributions of the polymer samples were calculated according to the Thomson–Gibbs equation:

*T*_m_ = *T*_m_°[1 − 2σ_e_/( *L* × ∆*H_f_*°)], (1)

where *T*_m_° is equilibrium melting temperature, *L* is lamellar thickness, σ_e_ is free surface energy of the end faces at which the chains fold, and ∆*H_f_*° is melting enthalpy of a perfect crystal. For calculation of the polyethylene samples, the following parameters were applied: *T*_m_° = 145.8 °C [47], σ_e_ = 90 × 10^−7^ J/cm^2^ [48], and ∆*H_f_*° = 289.4 J/cm^3^ [49]. For calculation of the polypropylene samples, the following parameters were applied: *T*_m_° = 208.0 °C [50], σ_e_ = 70 × 10^−7^ J/cm^2^ [51], and ∆*H_f_*° = 154 J/cm^3^ [52]. The results are shown in Figure 6.

The two series of polymers showed significantly different lamellar thickness distributions. Lamellar thickness of the PE samples was distributed in the size range of larger than 20 nm, with the peak values appearing at about 40 nm. However, the PP samples showed rather narrow lamellar thickness distributions, with the peak values appearing at about 9 nm. There were almost no lamellae thicker than 11 nm in the PP samples. By comparing with pore size distributions of the catalyst and polymer/catalyst particles (Figure 5), we can find that formation of PP lamellae inside the 20 nm pores of the catalyst particles is possible, but PE lamellae cannot grow inside these small pores because of their much larger thickness.

On the basis of the results of polymerization kinetics, solid structure of polymer/catalyst particles, and polymer aggregation state in the nascent particles, we can propose a mechanistic model to reasonably explain the distinct kinetic behaviors between ethylene and propylene polymerizations with the same supported Z–N catalyst, which is described in the following points:

1. Changes of active center concentration in the initial stage (0–10 min) clearly show that the lower rate of ethylene polymerization compared with that of propylene can be attributed to a much slower build-up of [C*] in the former system.
2. Both polymerization systems experienced a similar degree of diffusion limitation in the first 0–3 min, as shown by the larger apparent propagation rate constant in ethylene polymerization and similar slopes of the *k*_p_ versus *m*_P_/*m*_Cat_ curves. The porosity of PE/catalyst particles was larger than that of the PP/catalyst particles. The lower activity of ethylene polymerization cannot be attributed to its stronger diffusion limitation.
3. The catalyst particles have rather compact solid structure, though they are composed of sub-particles with a size of about 200–500 nm, and there are cracks with widths ranging from 100 nm to 5 µm. Pore size distribution, determined by the nitrogen adsorption method, shows that the nanometer pores in the catalyst are concentrated in the range of 15–25 nm. There is a huge number of such small pores, which renders the catalyst a very large specific surface area (282 m^2^/g) and high porosity (0.32 cm^3^/g). These 20 nm pores should be uniformly scattered in the solid phase of the catalyst. Assuming that the sub-particles are dense cubes with edges of 200 nm and a density of 2.34 g/cm^3^ (density of MgCl_2_ crystal), their aggregate will have a specific surface area of 13 m^2^/g, which is far lower than the measured specific surface area. The measured value of 282 m^2^/g will correspond to MgCl_2_ crystallite size (length of cube edges) of about 9 nm. This size is quite close to that of MgCl_2_ crystallites (7 nm) in supported Z–N catalysts determined by E. Redzic et al. [53]. Therefore, the 200–500 nm sub-particles cannot be dense solid, but rather aggregates of smaller MgCl_2_ crystallites containing many nanopores. It is likely that there is a large number of pores and cracks of about 20 nm in the sub-particles.
4. Because the sizes of PE lamellae formed by the growing polymer chains are far larger than the size of nanopores in the catalyst’s sub-particles, these lamellae cannot grow inside the nanopores, leaving the porous sub-particles basically intact during ethylene polymerization. Only the active sites exposed on the outer surface of the sub-particles can be activated and work as catalytic centers, but a large proportion of active site precursors is buried in the sub-particles and thus becomes unavailable to the polymerization reaction, resulting in a low [C*]/[Ti] ratio of ethylene polymerization. With the proceeding of polymerization, the PE layer covering the sub-particle will form a diffusion barrier that grows quickly with the increase of the *m*_P_/*m*_Cat_ ratio, and finally leads to ceasing of the polymerization.
5. In the propylene polymerization system, the PP lamellae with a size of 6–11 nm can enter the 20 nm pores in the sub-particles. Growth of these PP lamellae in the pores can exert hydraulic forces strong enough to break up the sub-particles and release their buried active site precursors. Subsequently, the 20 nm pores in the sub-particles will disappear, and the exposed surfaces carrying active sites will be covered by PP chains. After a short period of time, the whole polymer/catalyst particle will become rather compact, and the texture of the particle becomes rather smooth. Though the PP layer covering the MgCl_2_ crystallites (carrier of the active sites) can also cause serious diffusion barrier, for the much higher density of active sites in this system compared with the ethylene polymerization, the dynamically renewed carrier surface can allow for the presence of tiny pores in the PP layer. This will enable slow but stable diffusion of monomer stream in the PP layer, and a stable polymerization rate supported by a high [C*]/[Ti] ratio and low apparent rate constant.

This explanation is still far from comprehensive, but as we believe, it is the most reasonable one based on the present experimental results. According to this mechanism, fragmentation of the catalyst particles in the initial stage of polymerization is the decisive factor that determines the [C*]/[Ti] fraction, and thus the catalyst’s productivity, reachable in the main reaction stage. In the case of olefin polymerizations forming crystalline polymers, the difference between lamellar thickness of the polymer phase and pore size in the catalyst particle is also a key factor. Growth of thin polymer lamellae inside the nano-sized pores can cause further fragmentation of the sub-particles and exposure of more active sites. When the polymer chains form lamellae thicker than the pore size in the sub-particles, the lamellae tend to grow in space out of the nanopores in order to avoid the space confinement effects that require extra energy. It is thus expected that factors leading to reduction of lamellar thickness will promote fragmentation of the catalyst particles and enhance the catalytic activity. Enhancement of ethylene polymerization activity by prepolymerization with propylene can be explained by enhanced particle fragmentation in the prepolymerization stage. The comonomer activation effect in ethylene/α-olefin copolymerization [31] can also be explained by enhancement of particle fragmentation, because lamellar thickness of copolymer is evidently smaller than that of ethylene homopolymer [53,54]. On the other side, MgCl_2_-supported Z–N catalysts with average pore size far larger than 20 nm are found to have a rather high [C*]/[Ti] ratio in ethylene polymerization [23], or show ethylene polymerization activity much higher than the level reported in this work [55]. However, the presence of the soft amorphous phase (which is always present in PE or PP particles) in the nascent polymer will make the real mechanism more complicated than the simplified mechanism discussed here. J. Loos et al. found that catalyst fragmentation in propylene polymerization with the Z–N catalyst was slowed down by introducing ethylene as a comonomer [56]. It seems that the hard crystalline lamellae, for their low mobility, are more effective than the amorphous phase in expanding the nanopores and breaking the catalyst. More experimental and theoretical studies are expected to fully disclose the mechanism of initial polymerization kinetics and fragmentation of catalyst particles.

## 4. Conclusions

Homopolymerizations of ethylene and propylene with the same MgCl_2_-supported Z–N catalyst containing an internal electron donor showed remarkably different kinetic behaviors. Activity of ethylene polymerization was about 75% lower than that of propylene, and the polymerization rate quickly decayed with time in the former system, in contrast to a stable *R*_p_ versus time profile in the latter. The ethylene system showed a very low [C*]/[Ti] ratio (<0.6%) in the 10 min reaction process, in contrast to a much higher [C*]/[Ti] ratio (1.5%–4.9%) in propylene polymerization. The two systems showed noticeably different morphologies of the nascent polymer/catalyst particles, with the PP/catalyst particles being more compact and homogeneous than the PE/catalyst particles. On the basis of the observed phenomena, the different kinetic behaviors of the two reaction systems can be explained by faster and more sufficient catalyst fragmentation in propylene polymerization than in the ethylene system. In the propylene system, a large proportion of active sites was exposed through catalyst particle fragmentation in the polymerization process, but the degree of particle fragmentation was remarkably lower in the ethylene system. The larger thickness of PE lamellae in the nascent polymer than the average size of nano-pores in the catalyst is considered the main reason for the low efficiency of particle fragmentation in the ethylene system. In contrast, for the smaller lamellar thickness (<20 nm) of PP compared with the size of nanopores (15–25 nm) in the catalyst, PP lamellae may grow inside the nanopores and break up the catalyst particles by their hydraulic forces. The importance of catalyst fragmentation in fully releasing active site precursors in the catalyst and realizing high polymerization activity is clearly manifested in this work. Matching between the polymer’s lamellar thickness and size of the catalyst’s nanopores is an important factor that determines the efficiency of catalyst fragmentation.

## Figures and Tables

**Table 1:** Kinetic parameters and polymer properties of ethylene and propylene polymerizations *^a^*.

Run	*t*_p_*^b^* (s)	*m*_P_/*m*_Cat_*^c^* (g/g)	Activity (kg/g Ti·h)	*M*_w_*^d^* (10^5^)	*Đ ^d^*	*R*_p_ (10^−3^ mol/L·s)	[C*]/[Ti] (%)	*k*_p_ (L/mol·s)
E1	30	0.17	0.76	3.34	10.6	- *^e^*	- *^e^*	- *^e^*
E2	60	0.35	0.78	3.71	8.1	0.32	0.20	2035
E3	120	0.65	0.72	4.26	16.7	0.48	0.25	2410
E4	180	1.12	0.83	5.79	12.6	0.54	0.49	1380
E5	240	1.67	0.93	5.51	9.5	0.45	0.51	1103
E6	480	2.38	0.66	5.67	14.0	0.04	0.55	85
E7	600	2.39	0.53	6.21	10.8	0.01	0.59	17
P1	30	0.36	1.61	1.51	5.3	2.27	0.37	1433
P2	60	1.56	3.48	1.57	5.7	2.27	1.50	356
P3	120	5.44	6.05	1.59	6.2	2.27	3.10	172
P4	180	8.05	5.97	1.49	5.3	2.27	3.25	164
P5	240	11.17	6.20	1.24	5.6	2.27	3.35	160
P6	480	25.12	6.98	1.25	5.3	2.27	4.51	118
P7	600	30.34	6.74	1.27	5.5	2.27	4.87	110

*^a^*, polymerization conditions: runs E1–E7 were ethylene polymerization and P1–P7 were propylene polymerization; [Ti] = 1.0 mmol/L; triethylaluminum (TEA)/Ti = 40 (mol/mol); pressure of ethylene and propylene = 1 atm; polymerization temperature = 40 °C. Conditions of quench-labeling: 2-thiophenecarbonyl chloride (TPCC)/Al = 2.5 (mol/mol); quenching time = 5 min; *^b^*, duration of polymerization; *^c^*, yield of polymer based on unit catalyst weight; *^d^*, weight average molecular weight (*M*_w_) and polydispersity index (*Đ*); *^e^*, not determined because of insufficient sample weight.

**Table 2:** Structural parameters of catalyst and nascent polymer/catalyst particles.

Sample	Specific Surface Area (m^2^/g)	Total Pore Volume (cm^3^/g)	Average Pore Size (nm)
Cat.	281.55	0.320	22.37
E3	34.84	0.065	37.95
E4	51.85	0.090	33.36
P1	2.79	0.013	95.80
P2	1.55	0.009	117.44

**Table 3:** Thermal properties of nascent polymer particles.

Run	Polymer	*T*_m_*^a^* (°C)	∆*H*_f_ *^b^* (J/g)	*X*_c_*^c^* (%)
E1	PE	139.5	228.8	79.4
E2		140.1	208.5	72.4
E3		139.9	201.1	69.8
E4		141.8	200.7	69.7
P1	PP	161.0	86.7	56.3
P2		161.6	83.4	54.2
P3		161.0	79.8	51.8
P4		161.1	69.2	44.9

*^a^*, melting temperature; *^b^*, melting enthalpy; *^c^*, degree of crystallization calculated based on 100% defect free polyethylene crystal with a 289 J/g fusion heat [49] and polypropylene crystal with a 154 J/g fusion heat [52].

## Supplementary Materials

The following are available online at http://www.mdpi.com/2073-4360/11/2/358/s1. Figure S1: Changes of apparent rate constant with polymer/catalyst mass ratio; Figure S2: SEM pictures of catalyst particles; Figure S3: SEM pictures of polyethylene/catalyst particles at different polymerization times; Figure S4: SEM pictures of polypropylene/catalyst particles at different polymerization times; Figure S5: Nitrogen adsorption–desorption isotherms of polymer/catalyst particles; Figure S6: DSC curves of PE and PP samples (the first heating scan); Table S1: Thermal Properties of Polymer Samples Based on the Second Heating Scan.
