# Supplementary material for: Comparative Study on Kinetics of Ethylene and Propylene Polymerizations with Supported Ziegler–Natta Catalyst: Catalyst Fragmentation Promoted by Polymer Crystalline Lamellae
Source: Polymers (Basel). 2019 Feb 19;11(2):358. doi: 10.3390/polym11020358 (PMC6419229; doi:10.3390/polym11020358)
Supplement: Supplementary file 1 [file polymers-11-00358-s001.pdf]

Supporting Information

Comparative Study on Kinetics of Ethylene and Propylene Polymerizations with Supported Ziegler-Natta Catalyst Containing Internal Donor: Catalyst Fragmentation Promoted by Polymer Crystalline Lamellae

Zhen Zhang, Baiyu Jiang, Feng He, Zhisheng Fu, Junting Xu, Zhiqiang Fan*

MOE Key Laboratory of Macromolecular Synthesis and Functionalization, Department of Polymer Science and Engineering, Zhejiang University, Hangzhou 310027, China

Correspondence to: Z. Fan (E-mail: fanzq@zju.edu.cn)

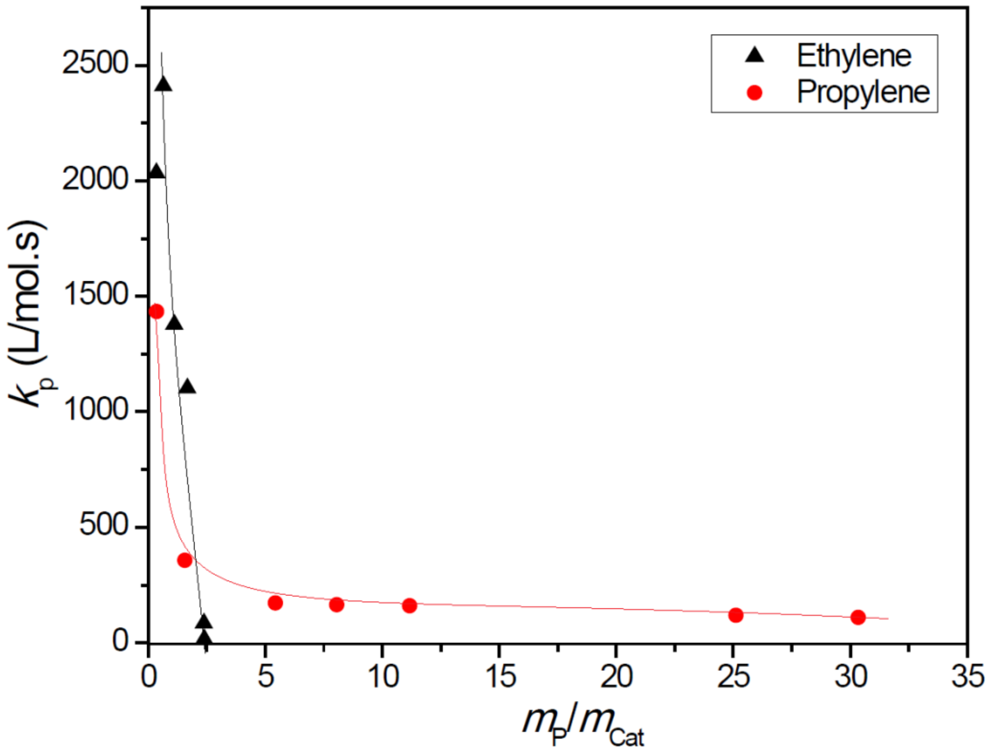

Figure S1. Changes of apparent rate constant with polymer/catalyst mass ratio.

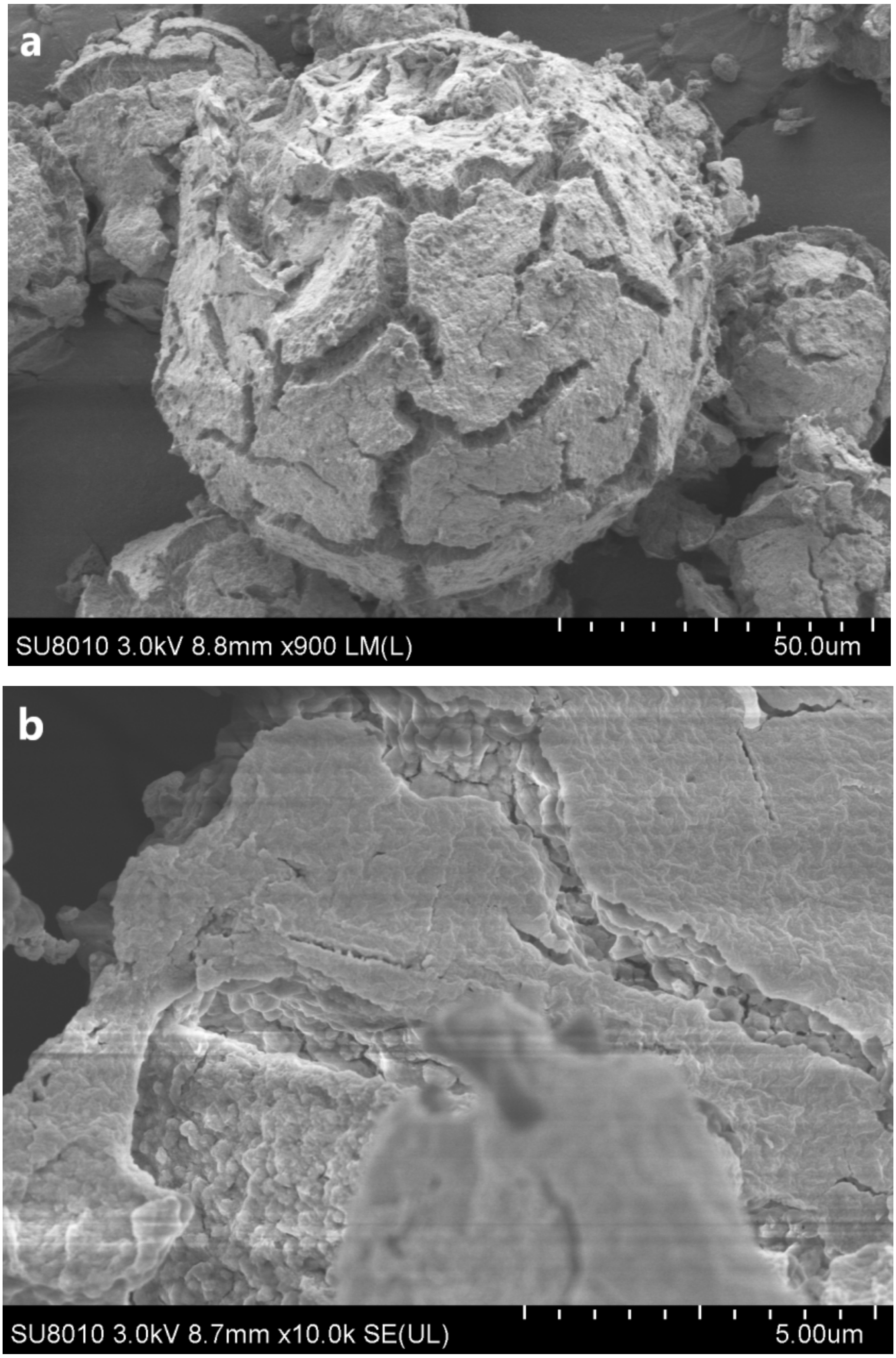

Figure S2. SEM pictures of catalyst particles.

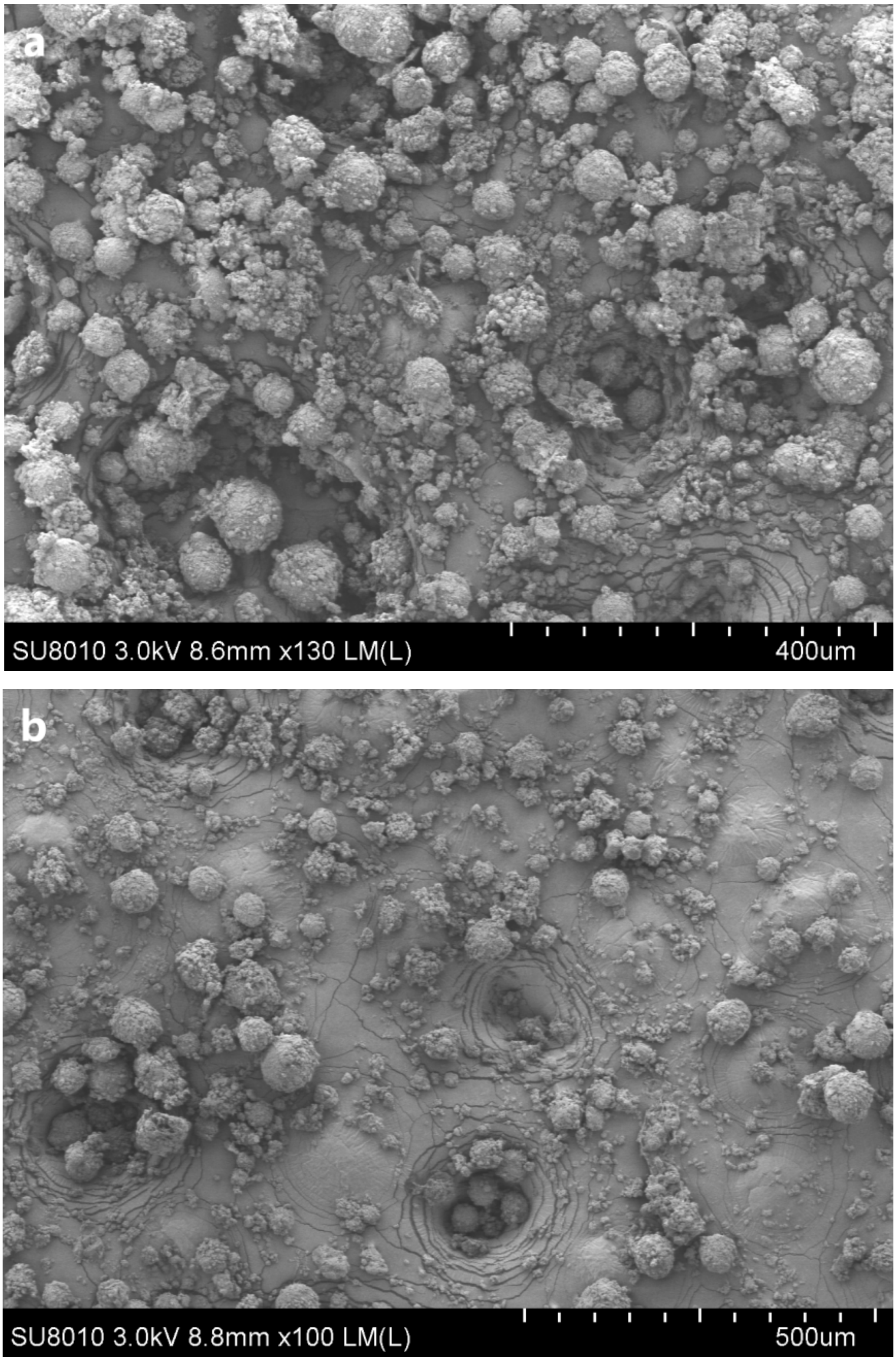

Figure S3. SEM pictures of polyethylene/catalyst particles at different polymerization times: (a) 120 s; (b) 180 s (samples E3 and E4 in Table 1).

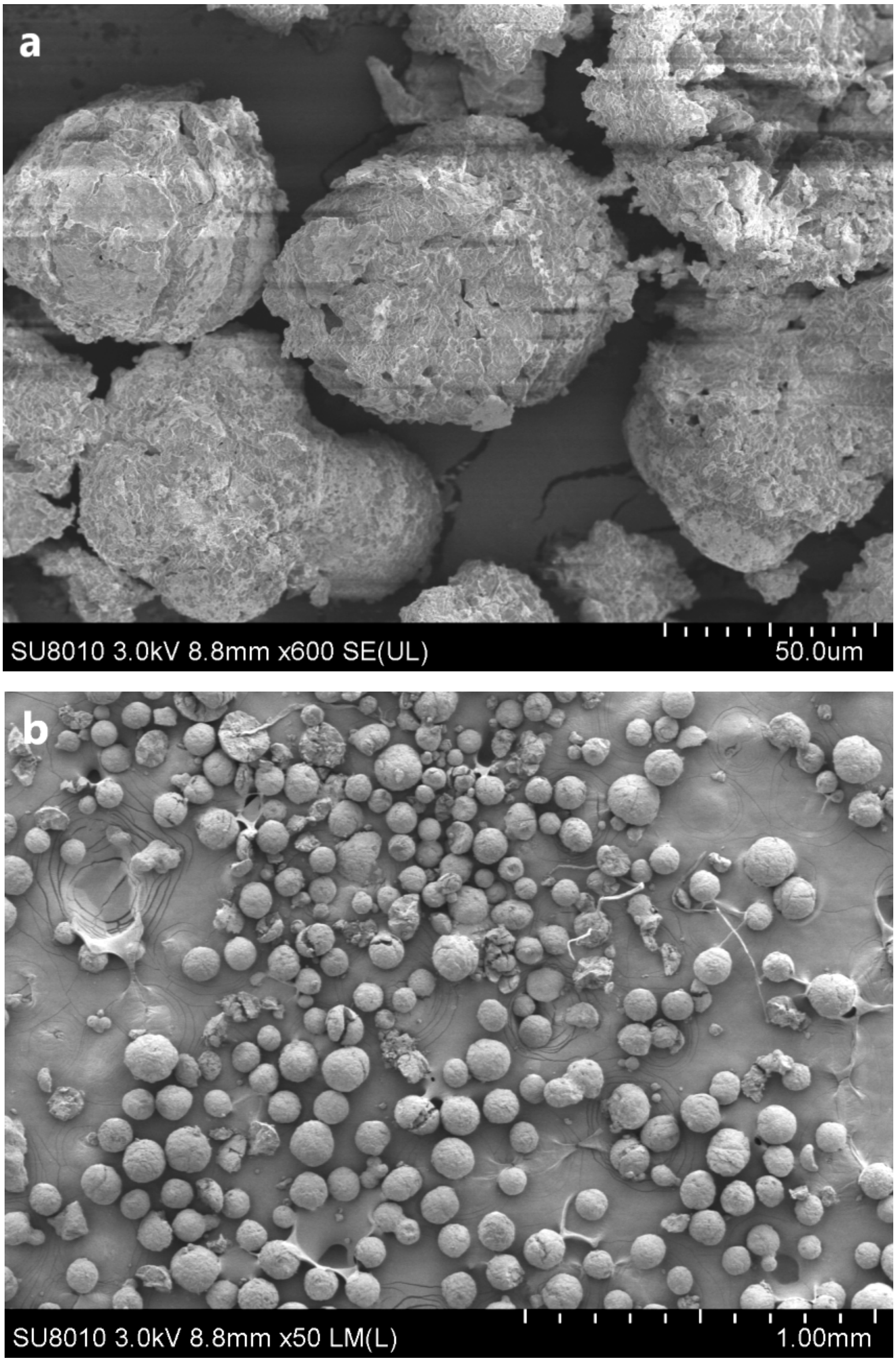
a
SU8010 3.0kV 8.8mm x600 SE(UL)
50.0um
b
SU8010 3.0kV 8.8mm x50 LM(L)
1.00mm

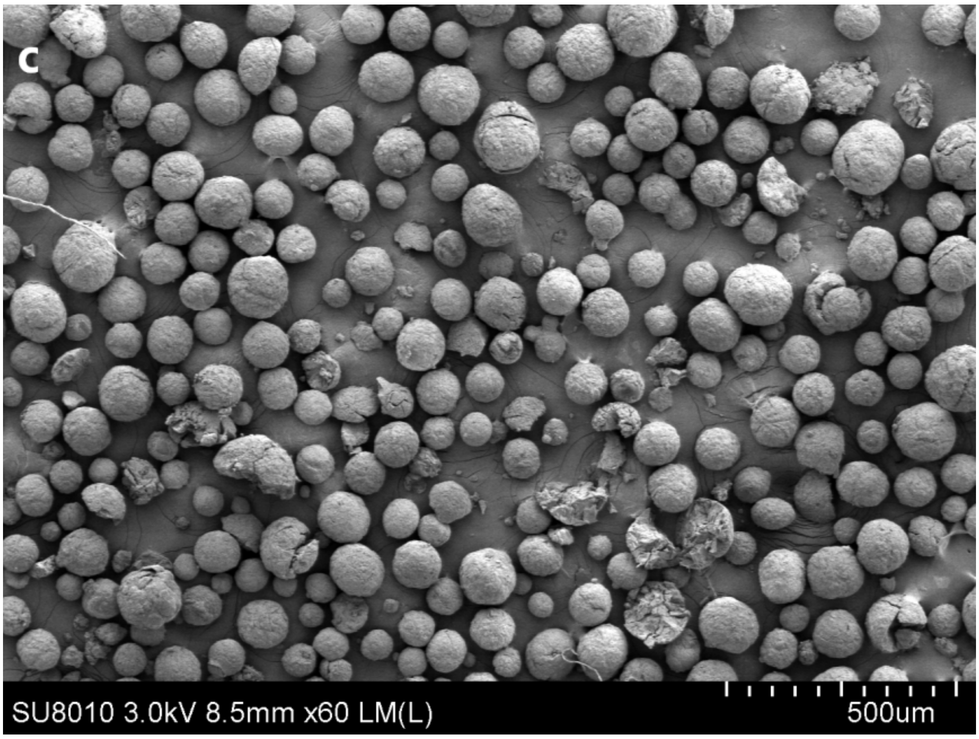

Figure S4. SEM pictures of polypropylene/catalyst particles at different polymerization times: (a) 30 s; (b) 120 s; (b) 180 s (samples P1, P3 and P4 in Table 1).

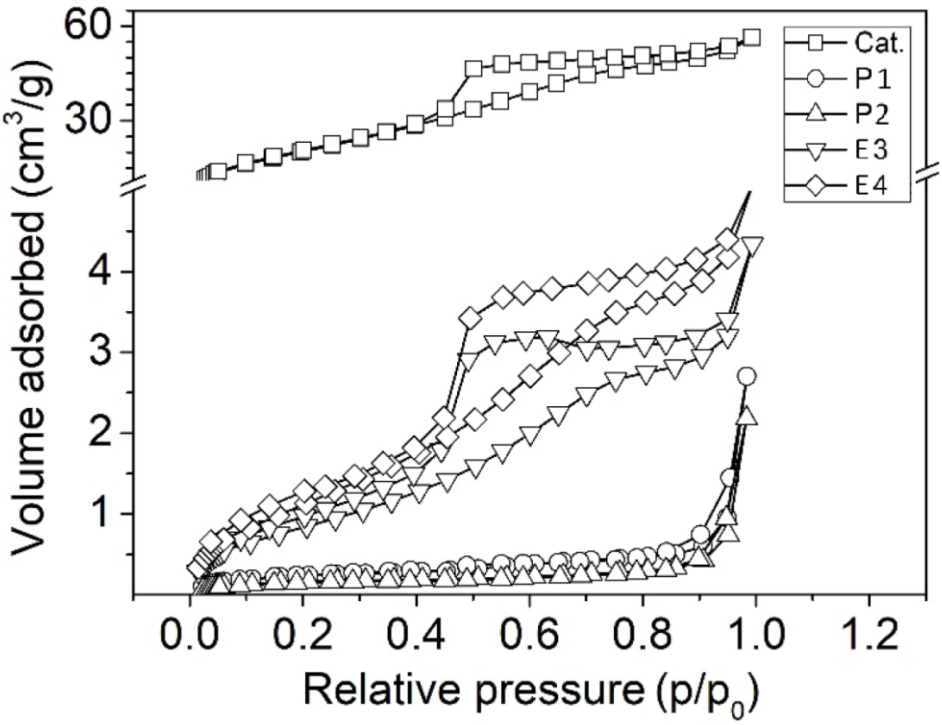

Figure S5. Nitrogen adsorption–desorption isotherms of polymer/catalyst particles.

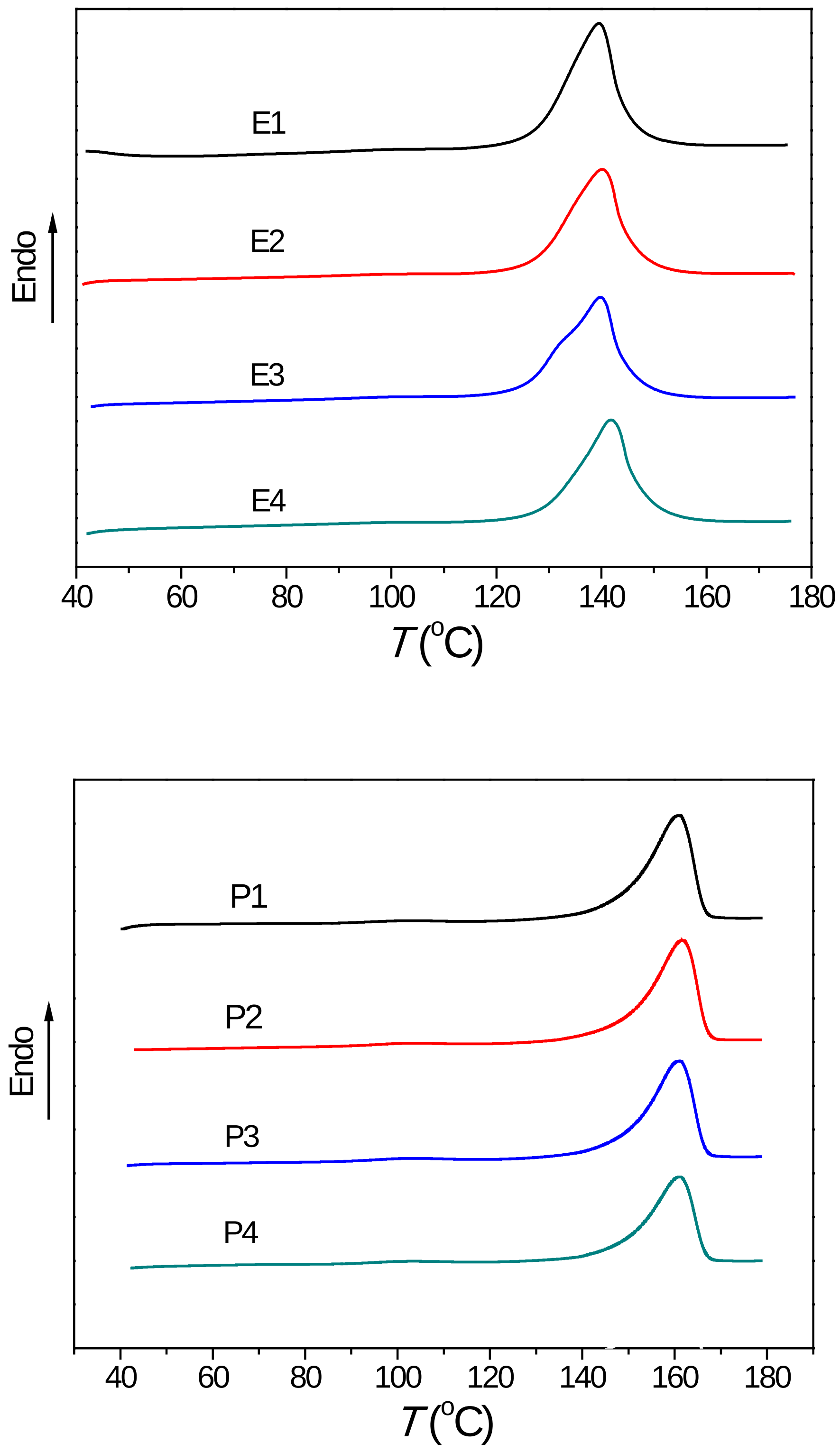

Figure S6. DSC curves of PE and PP samples (the first heating scan).

Table S1. Thermal Properties of Polymer Samples Based on the Second Heating Scan.

Run	Polymer	T_m [a] (°C)	ΔH_f [b] (J/g)	X_c [c] (%)
E1	PE	134.1	179.0	62.2
E2		134.9	164.3	57.0
E3		133.2	163.9	56.9
E4		133.8	142.0	49.3
P1	PP	159.5	78.5	51.0
P2		160.3	80.2	52.1
P3		158.8	78.3	50.8
P4		158.6	70.6	45.8

a. Melting temperature; b. Melting enthalpy; c. Degree of crystallization calculated based on 100% defectfree polyethylene crystal with a 288 J/g fusion heat and polypropylene crystal with a 154 J/g fusion heat.
